# Longitudinal association of circulating inflammatory biomarkers with epigenetic ageing in the Young Finns Study

**DOI:** 10.1038/s41598-026-46275-6

**Published:** 2026-03-31

**Authors:** Lauri Humaloja, Saara Marttila, Emma Raitoharju, Nina Mononen, Sirpa Jalkanen, Marko Salmi, Mika Kähönen, Olli T. Raitakari, Terho Lehtimäki, Pashupati P. Mishra

**Affiliations:** 1 Department of Clinical Chemistry, Faculty of Medicine and Health Technology, Tampere University, Tampere, Finland; 2 Finnish Cardiovascular Research Center Tampere, Faculty of Medicine and Health Technology, Tampere University, Tampere, Finland; 3 Department of Clinical Chemistry, Fimlab Laboratories, Tampere, Finland; 4 Molecular Epidemiology, Faculty of Medicine and Health Technology, Tampere University, Tampere, Finland; 5 Gerontology Research Center, Tampere University, Tampere, Finland; 6 Tays Research Services, Wellbeing Services County of Pirkanmaa, Tampere University Hospital, Tampere, Finland; 7 MediCity Research Laboratory, University of Turku, Turku, Finland; 8 Institute of Biomedicine, University of Turku, Turku, Finland; 9 Department of Clinical Physiology, Tampere University Hospital, Tampere, Finland; 10 Research Centre of Applied and Preventive Cardiovascular Medicine, University of Turku, Turku, Finland; 11 Department of Clinical Physiology and Nuclear Medicine, Turku University Hospital, Turku, Finland; 12 Centre for Population Health Research, University of Turku and Turku University Hospital, Turku, Finland; 13 InFLAMES Research Flagship, University of Turku, Turku, Finland; 14 Tampere University Hospital, Wellbeing Services county of Pirkanmaa, Turku, Finland

**Keywords:** DNA methylation, Epigenetic clock, Cytokines, Inflammation, Biological aging, Biomarkers, Diseases, Genetics, Medical research

## Abstract

DNA methylation-based epigenetic clocks are reliable measures of biological age and aging rate. Chronic inflammation may contribute to aging and various diseases, but population-based studies on specific inflammatory biomarkers’ impact on epigenetic clocks are limited. The aim of this study was to investigate the associations between 38 circulating inflammatory biomarkers, as well as a combined systemic inflammation variable, and epigenetic clocks in a middle-aged population. The cohort included 1,327 Finnish participants (aged 30–45 years, 50–55% female) from the Young Finns Study. Biomarkers were measured in 2007, and epigenetic clocks were assessed in 2011 and 2018. DunedinPACE and PCGrimAgeDev clocks were calculated using blood methylation data. Multiple linear regression models adjusted for age, sex, BMI, smoking, socioeconomic status, alcohol consumption, and physical activity were used. Results showed 11 biomarkers positively associated with DunedinPACE across both follow-ups. Seven biomarkers were positively associated with PCGrimAgeDev in the 4-year follow-up, but not in the 11-year follow-up. The combined systemic inflammation marker was positively associated with both clocks in both follow-ups. Although previous cross-sectional studies have reported associations between pro-inflammatory cytokines and epigenetic ageing, longitudinal findings remain sparse. Our results extend this literature by showing that several cytokines predict accelerated epigenetic ageing across an 11-year follow-up.

**Supplementary Information:**

The online version contains supplementary material available at 10.1038/s41598-026-46275-6.

## Introduction

Chronological age is a major predictor of functional decline, disease, and mortality. However, aging as a process begins before old age, and differences in the rate of biological aging may explain the variability in outcomes among elderly individuals^1,2^. The concept of biological aging is complex but is generally understood as the level of biological functioning of an organism, organ, or cell relative to chronological age^3^. Advances in aging research have identified 12 hallmarks of aging, including chronic inflammation and epigenetic alterations^4^. Various methods to quantify biological aging have been proposed, with epigenetic clocks emerging as some of the most promising and robust approaches^1,5^. Given that chronic inflammation is a hallmark of aging, an association between cytokines and epigenetic aging is to be expected^4^.

DNA methylation (DNAm) levels have been observed to change during aging^6^. The advent of DNA methylation arrays and sequencing technologies has enabled the determination of an individual’s methylome, facilitating the development of mathematical algorithms for estimating epigenetic age^7^. These DNAm age estimators, commonly referred to as epigenetic clocks, are used to measure both chronological and biological age. This study focuses on two epigenetic clocks recognized as strong predictors of mortality and among the best available measures of biological age: DunedinPACE^8^ and PCGrimAge^9,10^. DunedinPACE was developed using longitudinal organ-system integrity data from the Dunedin study. Researchers calculated each participant’s Pace of Aging and then applied elastic-net regression to develop a DNAm-based algorithm predicting Pace of Aging—this biomarker is termed DunedinPACE^8^. In contrast, GrimAge is a robust lifespan predictor that estimates mortality risk and biological age. It is defined as a weighted linear combination of DNAm surrogates for seven plasma proteins (ADM, B2M, cystatin C, GDF-15, leptin, PAI-1, TIMP1), a DNAm surrogate for smoking pack-years, as well as age and sex^10^. Due to the noisy nature of Illumina array methylation data, principal component (PC) algorithms have been developed to improve the reliability of GrimAge and other epigenetic clocks^9^. Epigenetic clocks have been associated with various diseases, including cardiovascular diseases, cancer, and metabolic syndrome, as well as numerous lifestyle factors, such as smoking, alcohol consumption, physical activity, nutrition, adiposity, education, and socioeconomic status^11^.

The immune system and inflammation play significant roles in the aging process, with chronic inflammation recognized as a hallmark of aging. Inflammation increases with age, a phenomenon known as inflammaging, which is characterized by elevated levels of several circulatory inflammatory markers^4^. However, whether inflammation causes aging or aging drives inflammation remains unclear^12^. Cytokines, protein-based molecules primarily produced by immune cells (e.g., T cells, B cells, and monocyte-macrophages), act as intercellular messengers, especially during inflammation^13^. Cytokines are often categorized based on their function as pro-inflammatory (e.g., IL-1, TNF-α) or anti-inflammatory (e.g., IL-10, TGF-β). However, cytokines exhibit pleiotropic effects, meaning their function depends on factors such as microenvironment and concentration^14^. Cytokines have been reported to exert both beneficial and detrimental effects on the aging process, depending on the context^15^.

Previous studies have identified some individual inflammatory biomarkers positively associated with epigenetic clocks cross-sectionally, though fewer longitudinal associations have been observed^16–19^. Additionally, systemic inflammation variables—combinations of multiple specific inflammatory markers—have been found to be positively associated with epigenetic aging cross-sectionally but not longitudinally^16,20^. Over 300 proteins with cytokine-like activity have been identified, displaying diverse functions in the immune system and across other organ systems^13^. Nonetheless, there is a lack of studies examining the longitudinal associations between individual inflammatory cytokine markers, their combinations, and different epigenetic clocks.

To address this gap, the primary objective of this study was to investigate the longitudinal associations between 38 specific inflammatory biomarkers, along with a combined systemic inflammation marker, and epigenetic clocks in a middle-aged population. This age group allows for the quantification of aging processes while the majority of the cohort remains free from aging-associated diseases.

## Materials and methods

### Study population

The study population originates from the Young Finns Study (YFS)^21^, a multicenter longitudinal study investigating cardiovascular risk factors from childhood to adulthood in Finland. YFS began in 1980 with 3,589 participants aged 3–18 years. Major follow-up assessments were conducted in 1983, 1986, 2001, 2007, 2011, and between 2018 and 2020^22^. For this study, the population was restricted to individuals with plasma cytokine concentrations measured in the 2007 follow-up and blood DNA methylome data available from the 2011 (4-year) and 2018 (11-year) follow-ups. The maximum number of participants included in analyses was 1,327 for 2011 and 1,097 for 2018, with each individual analysis encompassing over 1,000 subjects.

Alcohol consumption was assessed by asking participants to report their alcohol intake during the previous week, with one unit defined as 14 g of alcohol^23^. Physical activity was quantified using metabolic equivalents (METs), which represent an index derived from the frequency, intensity, and duration of physical activity, including leisure activities and commuting (MET hours/week). One MET corresponds to the energy expenditure of one kilocalorie per kilogram of body weight per hour at rest^24^. Smoking status was categorized as daily smoking, less frequent smoking, or nonsmoking. Socioeconomic status was evaluated based on participants’ education levels: comprehensive school, upper secondary school, or university.

The study was approved by the Ethics Committee of the Hospital District of Southwest Finland on 20 June 2017 (ETMK:68/1801/2017). All participants provided written informed consent, and the study was conducted in accordance with the Declaration of Helsinki.

### Measurement of inflammatory markers

The concentrations of 48 inflammatory biomarkers were measured from fasting serum samples collected in 2007. The analysis was conducted using Bio-Rad’s premixed Bio-Plex Pro Human Cytokine 27-plex Assay and 21-plex Assay kits on the Bio-Rad Bio-Plex 200 System. Certain cytokines were outside standard range, leading to missing values in the final dataset. Santalahti^25^, To address this issue, only cytokines with data available for over 1,000 subjects were included in the final analysis, ensuring adequate sample sizes for all evaluations, with the exception of CXCL12 that had sample size of 998 in the 11-year follow-up. Participants with invalid cytokine measurements for a given cytokine were excluded from analyses specific to that inflammatory biomarker. In total, 37 specific cytokines and CRP were analyzed. Logarithmic transformation was applied to skewed inflammatory biomarker concentration distributions (all except Platelet-derived growth factor-BB and IL-1β) to approximate a normal distribution, as assessed using histograms. Full details of the cytokine data are presented in Supplementary Table 1.

### Determination of epigenetic age and estimated immune cell proportions

Genome-wide DNA methylation (DNAm) levels from whole blood were measured using the Illumina Infinium MethylationEPIC BeadChip, following Illumina’s standard protocol^22^. A detailed description of the preprocessing and normalization of the DNAm data is provided in the Supplementary methods.

DunedinPACE scores were calculated using the algorithm provided by the developers. A DunedinPACE score of 1 indicates that an individual is aging biologically at the same rate as a calendar year, while a score below 1 reflects slower aging, and a score above 1 indicates faster aging^8^.

PCGrimAge scores, which estimate biological age in years, were determined using the enhanced principal component (PC) algorithm^9^. Epigenetic age deviation (PCGrimAgeDev) was derived from PCGrimAge scores by regressing them on chronological age and extracting the residuals^10^.

In the 4-year follow-up, the proportions of CD8+ and CD4+ T cells, natural killer cells, B cells, monocytes, and granulocytes in white blood cells were estimated using the reference-based Houseman method^26^ via the estimateCellCounts function from the minfi Bioconductor package in R^27^. At 11-years, cell proportions were estimated using the methodology implemented in the meffil R/Bioconductor package^28^.

### Statistical analysis

Population descriptives were calculated as means and standard deviations for continuous variables, such as age and BMI, and as percentages for categorical variables, such as socioeconomic status. Pairwise correlations among the inflammatory biomarkers were evalueted by calculating a Pearson correlation matrix.

Multiple linear regression models were employed to evaluate the associations between specific inflammatory cytokines and biological age variables, which were the primary outcomes of interest. Three models were utilized: model 1, adjusted only for chronological age and sex; model 2, which additionally accounted for BMI, smoking status, socioeconomic status, alcohol consumption, and physical activity (MET); and model 3, which included all previously mentioned covariates along with cell proportions. These adjustments were made to account for factors that could influence epigenetic age or cytokine levels. For the analyses at 11-years, an additional technical covariate, “batches,” was included to account for the two DNA methylation profiling batches and different versions of EPIC arrays.

After analyzing the associations between specific inflammatory biomarkers and epigenetic clocks in the 4-year follow-up, an exploratory combined variable for systemic inflammation was introduced. This variable was defined as an unweighted sum of log-transformed cytokine concentrations. Inflammatory markers with robust associations with both epigenetic clocks, and weak to minimal intercorrelations with each other, were included in this combined variable. The associations between this systemic inflammation variable and epigenetic clocks were analyzed using the same multiple linear regression models described earlier.

To address the issue of multiple testing, false discovery rate (FDR) correction was applied for each model and epigenetic clock separately. We did not apply a global correction across all clocks, models, and timepoints. FDR-adjusted p-value of less than 0.05 was considered statistically significant. All statistical analyses were performed using R version 4.3.2^29^.

## Results

### Study population and intercorrelations among studied inflammatory biomarkers

The study population, drawn from the YFS, comprised 1,327 and 1,124 participants at the 4-year and 11-year follow-ups, respectively. Women represented 54.9% and 49.9% of the participants, and the mean ages were 41.8 and 48.9 years, respectively. Additional baseline characteristics are presented in Table 1.

**Table 1:** Population characteristics of study participants.

Follow-up	2011 (*n* = 1327)	2018 (*n* = 1124)
Women	728 (54.9%)	561 (49.9%)
Age, yrs	41.8 ± 5.0	48.9 ± 5.0
Body mass index, kg/m^2^	26.5 ± 4.9	27.9 ± 5.3
Alcohol consumption, unit/day	0.80 ± 1.1	0.78 ± 1.2
Physical activity index (MET)	21.8 ± 21.3	19.4 ± 17.9
Daily smoking, %	170 (12.8%)	141 (12.5%)
Sosioeconomic status*		
Comprehensive school, %	44 (3.3%)	54 (4.8%)
Upper secondary school, %	888 (66.9%)	709 (63.0%)
University, %	330 (24.9%)	328 (29.2%)
DunedinPACE	0.94 ± 0.09	1.0 ± 0.11
PCGrimAge, yrs	55.2 ± 5.1	60.5 ± 4.9

Data are expressed as mean ± SD or percentages.

*Socioeconomic status based on education level.

**Fig. 1:**
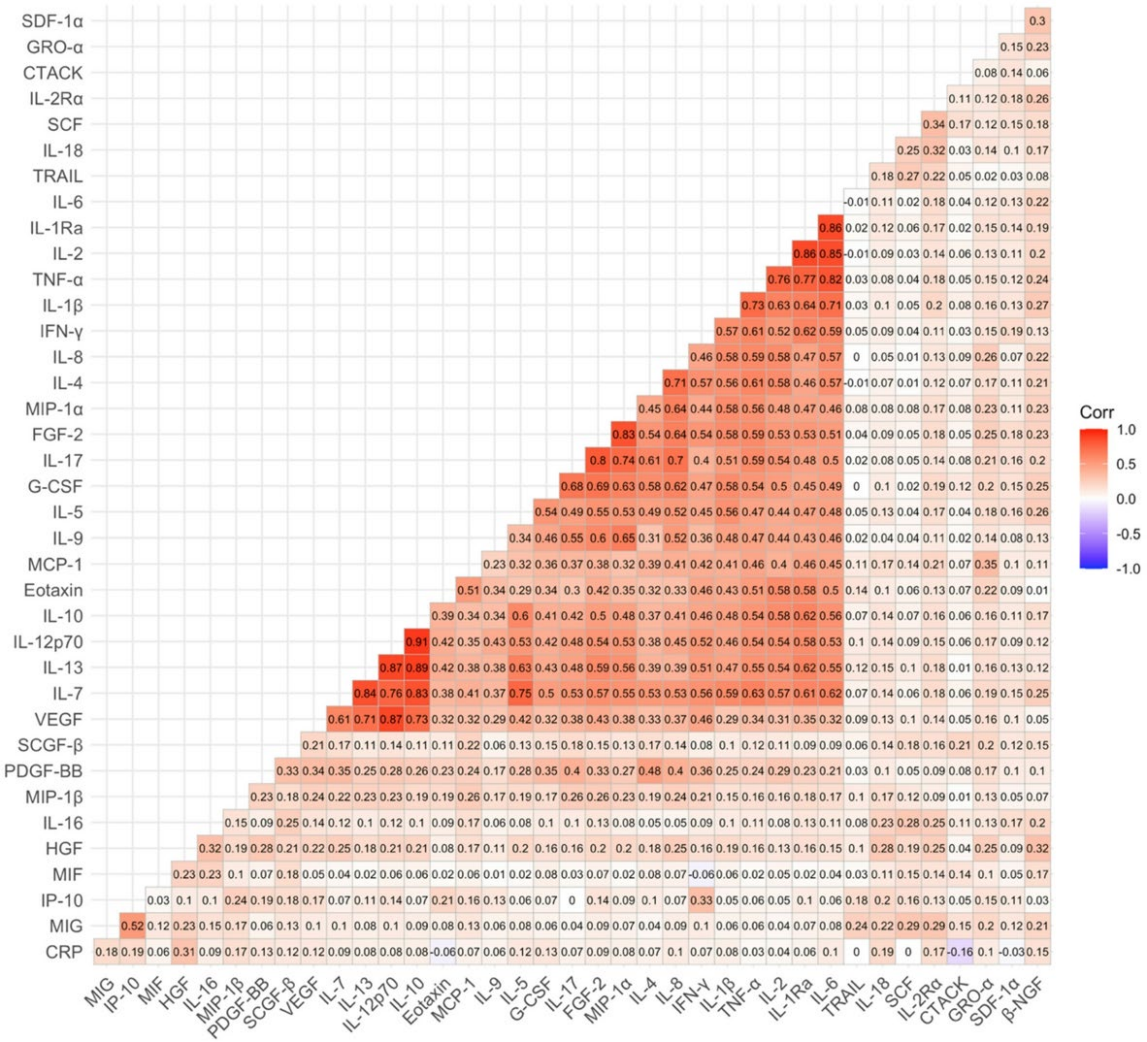
Correlation matrix of circulating inflammatory markers. Heatmap displaying pairwise Pearson correlation coefficients among all individual log-transformed* inflammatory markers that were included in the regression analysis. Color intensity reflects correlation strength, ranging from strong positive correlations (red) to negative correlations (blue). *All inflammatory biomarkers, except IL-1β and PDGF-BB, were log-transformed to better estimate a normal distribution.

Among the 38 inflammatory biomarkers studied, several positive pairwise correlations were observed (Fig. 1). A prominent cluster of interrelated cytokines, including Eotaxin, G-CSF, and IL-5, exhibited moderate-to-strong intercorrelations, whereas several other inflammatory biomarkers, such as IL-18, IL-2Rα, and HGF, showed weak to minimal correlations with other biomarkers. Additionally, CRP exhibited only weak or minimal correlations with the other inflammatory biomarkers.

### Longitudinal associations between inflammatory biomarkers and DunedinPACE

**Fig. 2:**
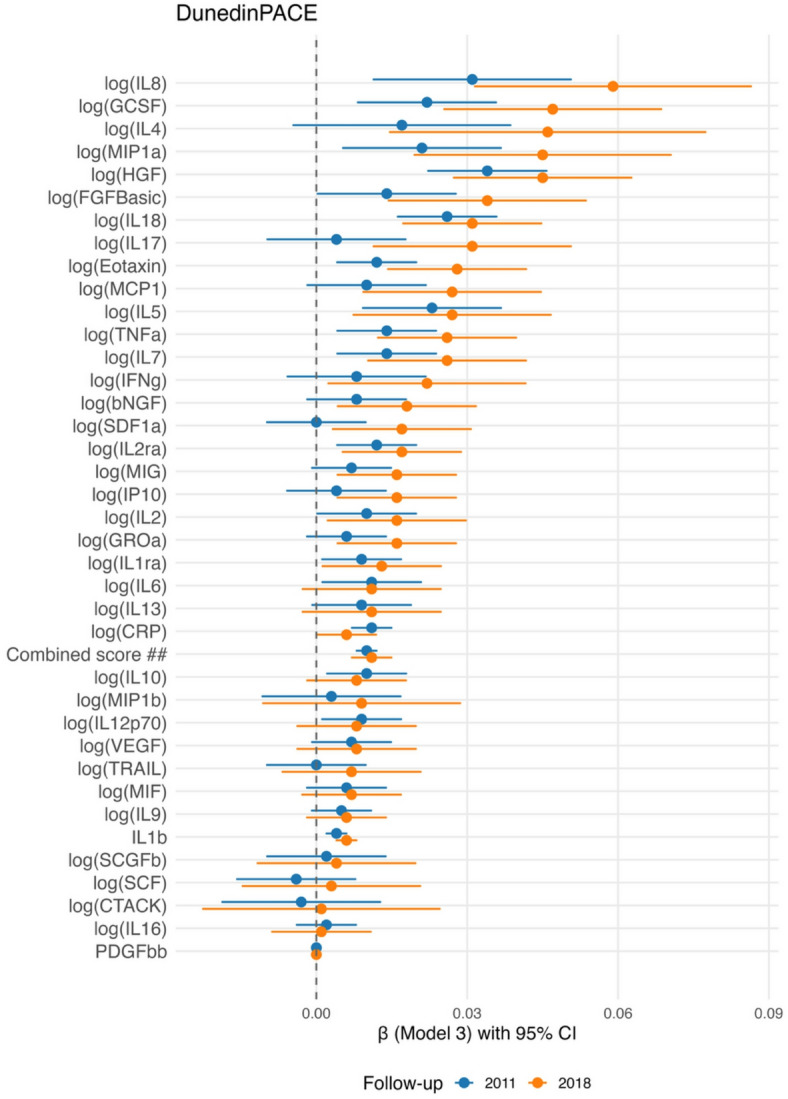
Longitudinal associations between inflammatory biomarkers and DunedinPACE. Forest plot showing regression coefficients in the most adjusted model and 95% confidence intervals for associations between inflammatory biomarkers from the 2007 follow-up (y-axis) and DunedinPACE (x-axis). Estimates are shown separately for the 2011 follow-up (blue) and the 2018 follow-up (orange). *Covariates in most adjusted model (model 3): chronological age, sex, BMI, socioeconomic status, alcohol use, smoking, physical activity (MET), and immune cell proportions. 2018 analyses adjusted additionally for a technical covariate “batches”. ## **Combined variable** is a sum of five log-transformed inflammatory biomarkers (CRP, Eotaxin, IL-18, IL2-rα, HGF).

After analyzing 38 inflammatory biomarkers using three different multiple linear regression models with varying covariate sets, 13 specific inflammatory biomarkers measured during the 2007 follow-up were found to be robustly associated with DunedinPACE in the 4-year follow-up (2011), while 16 biomarkers were associated in the 11-year follow-up (2018). Of these, 11 biomarkers exhibited consistent associations across both follow-ups. The results of the most adjusted model, with DunedinPACE as the outcome, are presented in a forest plot in Fig. 2. The results of all three regression models, with DunedinPACE as the outcome variable in the 4- and 11-year follow-ups, are provided in Supplementary Table 2.

In both the 4-year and 11-year follow-ups, nine cytokines—Eotaxin, GCSF, IL-18, IL-5, IL-7, IL-8, HGF, IL-1β, and IL-2rα—were found to be positively and statistically significantly associated with DunedinPACE in all three regression models. CRP demonstrated positive associations with DunedinPACE across all models in the 4-year follow-up. In the 11-year follow-up, CRP was positively associated in models 1 and 2 and showed borderline significance in model 3 (Adj. P-value = 0.065). MIP1a exhibited positive associations in models 1 and 3 and borderline significance in model 2 (Adj. P-value = 0.055) in the 4-year follow-up, while it was positively associated across all models in the 11-year follow-up. TNF-α was positively associated in all models at 4-years and in models 2 and 3 at 11-years.

The anti-inflammatory cytokine IL-10 was positively associated across all models in 4-year follow-up, but did not show significant associations in the 11-year follow-up. IL-12 was positively associated in 4-year follow-up in models 1 and 2, with borderline significance in model 3 (Adj. P-value = 0.056); however, no significant associations with IL-12 were observed at 11-years in any model. IL-17, MCP-1, MIG, and IP-10 were positively associated with DunedinPACE in all three regression models in the 11-year follow-up only.

### Longitudinal associations between inflammatory biomarkers and PCGrimAgeDev

**Fig. 3:**
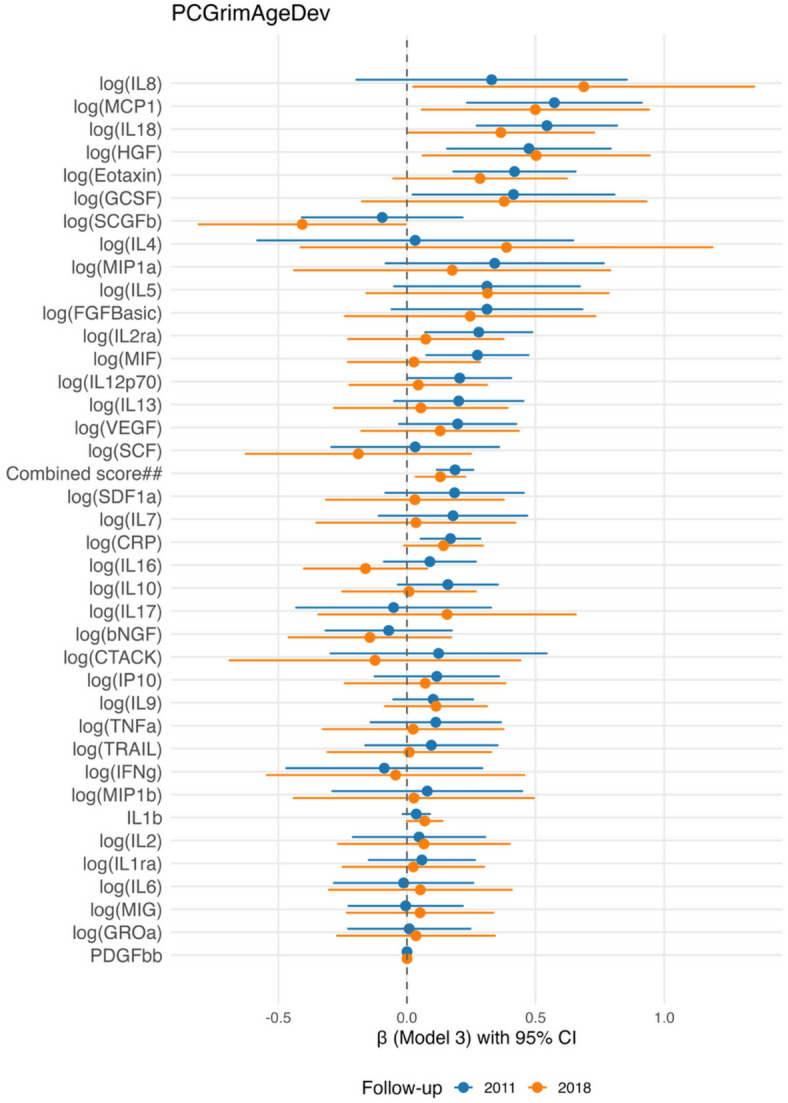
Longitudinal associations between inflammatory biomarkers and PCGrimAgeDev. Forest plot showing regression coefficients in the most adjusted model* and 95% confidence intervals for associations between inflammatory biomarkers from the 2007 follow-up (y-axis) and PCGrimAgeDev (x-axis). Estimates are shown separately for the **2011 follow-up (blue)** and the **2018 follow-up (orange).** *Covariates in most adjusted model (model 3): chronological age, sex, BMI, socioeconomic status, alcohol use, smoking, physical activity (MET), and immune cell proportions. 2018 analyses adjusted additionally for a technical covariate “batches”. ## **Combined variable** is a sum of five log-transformed inflammatory biomarkers (CRP, Eotaxin, IL-18, IL2-rα, HGF).

The results of the most adjusted model, with PCGrimAgeDev as the outcome, are presented in a forest plot in Fig. 3. The results for all 38 cytokines analysed using three differently adjusted multiple linear regression models, with PCGrimAgeDev as the outcome variable in the 4- and 11-year follow-ups, are presented in Supplementary Table 3.

The same three adjusted linear regression models used for DunedinPACE were applied to evaluate the longitudinal associations between inflammatory biomarkers and PCGrimAgeDev as the endpoint. Seven inflammatory biomarkers were found to be positively associated with PCGrimAgeDev in all regression models during the 4-year follow-up. However, no such systematic associations were observed across the three regression models in the 11-year follow-up.

In the 4-year follow-up, CRP was positively associated with PCGrimAgeDev in models 1 and 3, and it showed borderline significance in model 2 (Adj. P-value = 0.051). Eotaxin, IL-18, MCP-1, and the anti-inflammatory marker HGF were positively associated with PCGrimAgeDev in all regression models during the 4-year follow-up. IL-2rα was positively associated in models 1 and 3, while showing borderline significance in model 2 (Adj. P-value = 0.084).

In the 11-year follow-up, CRP, IL-18, MCP-1, VEGF, and the anti-inflammatory marker HGF were significantly and positively associated with PCGrimAgeDev in model (1) Additionally, in the 11-year follow-up, HGF was positively associated with PCGrimAgeDev in model (2) However, after applying the FDR correction, no specific inflammatory biomarker remained statistically significant in model 3 during the 11-year follow-up (see Supplementary Table 3).

### Combined variable of systemic inflammation and epigenetic clocks

Based on the analysis of associations between specific inflammatory biomarkers and epigenetic clocks from the 4-year follow-up, we constructed an exploratory combined variable estimating systemic inflammation in relation epigenetic ageing. This variable consisted of inflammatory markers that demonstrated robust associations with both epigenetic clocks during the 4-year follow-up. The five selected biomarkers included in the systemic inflammation variable were CRP, Eotaxin, IL-18, IL-2rα, and the anti-inflammatory marker HGF. Furthermore, these inflammatory markers exhibited weak or minimal intercorrelations with each other (Fig. 1). Among these markers, IL-18–IL-2rα and CRP–HGF demonstrated weak positive correlations (r_Pearson_= 0.32 and r_Pearson_ = 0.31, respectively), whereas the remaining pairwise correlations were below 0.3, consistent with little to no shared variance.

We also analysed the association of the combined variable of systemic inflammation with epigenetic clocks. The results from the most adjusted model are presented in Figs. 2 and 3. Detailed results for the combined systemic inflammation variable, analysed across three differently adjusted linear regression models, with either DunedinPACE or PCGrimAgeDev as the outcome variable, are presented in Supplementary Tables 2 and Supplementary Table 3 for the 4- and 11-year follow-ups. The combined variable was positively associated with both epigenetic clocks in the 4-year and 11-year follow-ups. For DunedinPACE, positive associations were observed in both follow-ups across all three regression models. Similarly, the combined variable showed positive associations with PCGrimAgeDev in the 4-year follow-up across all models. However, in the 11-year follow-up, the association with PCGrimAgeDev was statistically significant only in models 1 and 2 after applying the FDR correction.

## Discussion

Our study advances current knowledge by demonstrating, in a longitudinal, population-based setting, the association of seven novel cytokines with epigenetic aging, thereby introducing new candidates for the regulation of epigenetic age. Synthesizing our findings with prior research poses challenges due to several factors. Previous studies have examined diverse sets of inflammatory biomarkers, employed different epigenetic clock algorithms (both older and newer versions), and utilized various DNA methylation analysis platforms across non-population-based and population-based settings. Additionally, many earlier studies have focused solely on cross-sectional associations, whereas our study adopts a longitudinal design.

Nevertheless, our results align with the broader observation that (pro-)inflammatory markers are positively associated with epigenetic aging. For instance, we found CRP to be positively linked with DunedinPACE and PCGrimAgeDev, consistent with prior research showing cross-sectional positive associations with extrinsic epigenetic age acceleration (EEAA)^19,30^ and longitudinal associations with DunedinPoAm, an earlier version of DunedinPACE^16,31^. Similarly, the pro-inflammatory cytokine TNF-α was positively associated with DunedinPACE in our study, in line with previous cross-sectional findings linking it to EEAA^18^ and GrimAge^16^. We also observed that pro-inflammatory IL-18 was positively associated with both DunedinPACE and PCGrimAgeDev, consistent with prior cross-sectional evidence tying it to EEAA^17^. Likewise, IL-8 showed a positive association with DunedinPACE in our study, mirroring earlier cross-sectional associations with DunedinPoAm and GrimAge^16^. IL-2rα was positively associated with both DunedinPACE and PCGrimAgeDev in our findings, though a previous study reported a weak negative cross-sectional association with Horvath age acceleration^18^. We also examined IL-6, which prior studies have widely linked to various epigenetic clocks both cross-sectionally and longitudinally^16,18,19^. Surprisingly, in our longitudinal study, we found no statistically significant association with either epigenetic clock.

Other pro-inflammatory cytokines identified in our study (Eotaxin, IL-1β, GCSF, IL-5, IL-7, and MIP-1α) have not previously been investigated in the context of epigenetic aging, thus enriching our understanding of the role of inflammatory factors in this process. Among anti-inflammatory cytokines, HGF stood out as the only one robustly and positively associated with both epigenetic clocks in our study; it has not been previously explored in relation to epigenetic aging. While anti-inflammatory cytokines have received less attention than their pro-inflammatory counterparts in this field, and our study too primarily focused on pro-inflammatory markers, the identification of HGF suggests a need for further exploration of anti-inflammatory factors in epigenetic aging.

While previous research has established broad cross-sectional associations between circulatory inflammatory biomarkers and epigenetic aging, longitudinal studies on this topic have been limited. Cribb et al., conducted the only study assessing longitudinal relationships between a wide range of inflammatory biomarkers (22 in total) and second-generation epigenetic clocks. However, selection bias during the follow-up phase may have led to an underestimation of the association between inflammation and epigenetic aging^16^. Similarly, Stevenson et al., included a longitudinal analysis in their study but focused solely on two inflammatory markers, CRP and IL-6, in relation to first-generation epigenetic clocks^19^. In our study, we identified numerous longitudinal associations using two different time intervals, four and 11 years. Our findings suggest that pro-inflammatory cytokines are longitudinally associated with epigenetic aging, particularly with DunedinPACE, over a period of up to 11 years.

Additionally, we constructed a combined variable to represent systemic inflammation, comprising CRP, Eotaxin, IL-18, HGF, and IL-2rα, based on their robust associations with both DunedinPACE and PCGrimAgeDev in the 4-year follow-up. Importantly, these inflammatory biomarkers demonstrated weak to minimal intercorrelations with each other, indicating they represent relatively independent inflammatory processes. This variable demonstrated longitudinal associations with both DunedinPACE and PCGrimAgeDev across the four- and 11-year follow-ups. This supports the notion that systemic inflammation may contribute to accelerated ageing through several, relatively independent inflammatory pathways.

Previous researchers have also studied variables estimating systemic inflammation in relation to epigenetic clocks. Cribb et al., also studied an “inflammaging signature”^32^ and observed cross-sectional associations with epigenetic clocks, including GrimAge and DunedinPoAm, but not longitudinal ones^16^. Meier et al., investigated a latent (i.e., unobservable) variable of systemic inflammation and found cross-sectional associations with epigenetic clocks. However, they did not analyze longitudinal relationships with epigenetic aging. Interestingly, their latent variable was a better predictor of four-year mortality than the epigenetic clocks used in their study^20^. The systemic inflammatory variables described in these studies differ in their composition and methods of construction, making direct comparisons challenging. They may also reflect distinct underlying phenomena. Nevertheless, systemic inflammatory variables combining multiple markers can offer valuable insights into the unmeasured, underlying construct of systemic inflammation^20^. Considering our findings alongside previous research, it becomes evident that systemic inflammation, broadly defined as a combination of various inflammatory markers, is associated with accelerated epigenetic aging in both cross-sectional and longitudinal contexts.

Our comparison of the 4-year and 11-year follow-up data indicates that the association between inflammatory markers and DunedinPACE strengthens over time. In contrast, the association with PCGrimAgeDev appears to weaken. This attenuation may partly result from the slight reduction in sample size, which could explain the lack of statistical significance in the 11-year follow-up for PCGrimAgeDev. Additionally, the design differences between these epigenetic clocks may account for why DunedinPACE shows broader associations with inflammatory markers compared to PCGrimAgeDev. DunedinPACE is constructed based on longitudinal organ-system data, including CRP, making it more robust for estimating biological age^8^. Furthermore, because CRP is incorporated into the Pace of Ageing measure used to derive DunedinPACE, this clock may more accurately capture the inflammatory component of biological ageing, making it more likely to associate with inflammatory biomarkers than PCGrimAgeDev. Therefore, if inflammatory markers are indeed associated with biological aging, they are more likely to correlate with DunedinPACE than with PCGrimAgeDev, which functions primarily as a mortality predictor developed using cross-sectional data^9,10^.

Chronic inflammation is a hallmark of aging^4^. While inflammaging is a well-documented phenomenon, the exact direction of causality remains unclear—whether inflammation drives aging or vice versa^12^. Furthermore, it has been suggested that individual cytokines, which are messenger molecules of the immune system, play dual roles in aging. Depending on the context, the same cytokine may have either beneficial effects, such as promoting autophagy, or harmful effects, such as increasing oxidative stress, in the aging process^15^. In our study, pro-inflammatory cytokines were found to be longitudinally associated with epigenetic clocks, particularly DunedinPACE, in a cohort largely free of aging-related diseases. This finding aligns with the hypothesis that inflammation contributes to biological aging, though the process may function as a self-perpetuating cycle. While our results suggest that pro-inflammatory cytokines are associated with accelerated biological aging at a population level, they do not establish causality.

Our study possesses several notable strengths and novelties. First, we analyzed numerous inflammatory biomarkers that have not been previously investigated in relation to epigenetic age. Furthermore, the third-generation epigenetic clock, DunedinPACE, has not been previously studied in relation to inflammatory mediators. Additionally, there is a scarcity of representative studies on the longitudinal impact of inflammation on epigenetic aging, and our work makes a valuable contribution to this area of research. Second, we utilized a relatively young cohort, enabling us to explore the effects of inflammation on epigenetic aging in younger, healthier individuals who are free from aging-associated diseases. Third, our analyses were adjusted for a range of potential confounders, including immune cell proportions estimated from methylation data. Previous studies have identified immune cell composition as being associated with epigenetic clocks, such as DunedinPACE and GrimAge^33,34^. Researchers have recommended accounting for immune cell composition in analyses of epigenetic age deviation^34^. Moreover, we hypothesize that cytokine concentrations may be linked to cell proportions. Thus, adjusting for cell proportions significantly enhances the validity of our findings.

However, our study also has limitations. The cohort consists exclusively of Finnish individuals, which may restrict the generalizability of our findings to other populations. Additionally, there may be underlying confounders, such as metabolic health, that were not accounted for, potentially skewing our results. Lastly, due to data use restrictions, we were not able to use other prominent, third generation epigenetic clocks, such as GrimAge2^35^, and Systems Age^36^, which poses another limitation for our results.

## Summary and conclusions

Our findings advance current knowledge by identifying seven new cytokines associated with epigenetic aging—Eotaxin, IL-1β, GCSF, IL-5, IL-7, MIP-1a, and HGF. We present robust evidence that several pro-inflammatory and a few anti-inflammatory cytokines are positively associated with accelerated epigenetic aging, as assessed by DunedinPACE and PCGrimAgeDev over 4- and 11-year longitudinal follow-ups. Both pro- and anti-inflammatory cytokines showed positive associations with epigenetic clocks, which is unsurprising given the inherently complex and multifaceted nature of the biological mechanisms underlying inflammation. Furthermore, our results reveal that composite variables comprising multiple specific inflammatory biomarkers are longitudinally associated with epigenetic clocks, rather than merely cross-sectionally. Our findings suggest that several specific pro-inflammatory cytokines are linked to accelerated biological aging at a population level, even though individual cytokines may have dual roles in aging processes, depending on the context^15^.

Future research should prioritize replicating the novel discoveries of this study in diverse populations, further investigating the role of anti-inflammatory cytokines in relation to epigenetic clocks and elucidating the biological mechanisms driving the associations between inflammatory mediators and epigenetic aging. Unravelling these mechanisms could provide invaluable insights into the intricate relationship between inflammation and biological aging. Lastly, future studies should seek to study newer epigenetic clocks, such as GrimAge2^35^ and Systems Age^36^ in relation to inflammatory biomarkers to further elucidate the interplay between cytokines and biological ageing.

## Supplementary Information

Below is the link to the electronic supplementary material.

Supplementary Material 1

## Data Availability

The dataset supporting the conclusions of this article were obtained from the Cardiovascular Risk in Young Finns study which comprises health related participant data. The use of data is restricted under the regulations on professional secrecy (Act on the Openness of Government Activities, 612/1999) and on sensitive personal data (Personal Data Act, 523/1999, implementing the EU data protection directive 95/46/EC). Due to these restrictions, the data cannot be stored in public repositories or otherwise made publicly available. Data access may be permitted on a case-by-case basis upon request only. Data sharing outside the group is done in collaboration with YFS group and requires a data-sharing agreement. Investigators can submit an expression of interest to the chairman of the publication committee, Prof Olli Raitakari (University of Turku, Finland), Prof Mika Kähönen (Tampere University, Finland) and Prof Terho Lehtimäki (Tampere University, Finland). Requests to access these datasets should be directed to OR, olli.raitakari@utu.fi; TL, terho.lehtimaki@tuni.fi; MK, mika.kahonen@tuni.fi.
