# Supplementary material for: Longitudinal association of circulating inflammatory biomarkers with epigenetic ageing in the Young Finns Study
Source: Sci Rep. 2026 Mar 31;16:15543. doi: 10.1038/s41598-026-46275-6 (PMC13187333; doi:10.1038/s41598-026-46275-6)
Supplement: Supplementary file 1 — Supplementary Material 1 [file 41598_2026_46275_MOESM1_ESM.docx]

**Longitudinal association of circulating inflammatory biomarkers with epigenetic ageing in the Young Finns Study: supplementary material**

Table of Contents

[Supplementary methods 2](#_Toc224286265)

[Preprocessing and normalization of the 2011 methylation data 2](#_Toc224286266)

[Preprocessing and normalization of the 2018 methylation data 2](#_Toc224286267)

[Supplementary results 4](#_Toc224286268)

[Supplementary Table 1. Characteristics of the cytokine data. 4](#_Toc224286269)

[Supplementary Table 2. Longitudinal associations between inflammatory biomarkers and DunedinPACE 9](#_Toc224286270)

[Supplementary Table 3. Longitudinal associations between inflammatory biomarkers and PCGrimAgeDev 13](#_Toc224286271)

[References 18](#_Toc224286272)

# **Supplementary methods**

## **Preprocessing and normalization of the 2011 methylation data**

Initial quality control of samples was performed using the *minfi* package in R [Martin et al., 2014]. Multiple criteria were applied to ensure high quality data prior to downstream analyses. First, detection p-values were assessed to evaluate probe performance within each sample. Samples were retained if the mean detection p-value across all probes was less than 0.05 (i.e., colMeans(detP) < 0.05). Samples exceeding this threshold were excluded from further analysis. Second, signal intensity distributions were examined using the *getQC* function in *minfi*. Samples were expected to cluster based on the log2 median intensities of methylated and unmethylated signals. Samples that did not cluster with the main cohort were considered outliers and removed. Third, sex prediction was performed using the *getSex* function in *minfi*. Predicted sex was compared to reported sex, and samples with discordant sex assignments were excluded.

Background correction and normalisation were performed sequentially using the *preprocessNoob* and *preprocessQuantile* functions implemented in *minfi.* The *preprocessNoob* function was applied for background subtraction and dye-bias correction using the Normal-exponential Out-Of-Band (Noob) method, as described by [Timothy et al., 2013]. This method estimates background signal from out-of-band probes and corrects each sample individually. Dye-bias normalisation is simultaneously performed using a subset of control probes to estimate and adjust for dye-related technical variation. By default, both procedures are carried out within the function. Following background correction, stratified quantile normalisation was applied using *preprocessQuantile*. This approach performs both within and between sample normalisation. Because DNA methylation levels vary across genomic regions, stratification ensures that probes with similar characteristics are normalised together, thereby preserving biological variation while reducing technical variability.

After sample level quality control and normalisation, probe-level filtering was conducted. First, probes were retained only if they exhibited a detection p-value < 0.01 in at least 99% of the samples. Probes failing this criterion were removed. Second, probes located on sex chromosomes were excluded to avoid confounding effects related to sex-specific methylation differences. Third, probes containing single nucleotide polymorphisms (SNPs) at the CpG interrogation site were removed to minimise potential bias in methylation measurement. Finally, cross-reactive probes, those known to hybridise to multiple genomic locations, were excluded to ensure specificity of the methylation signal.

## **Preprocessing and normalization of the 2018 methylation data**

Genome-wide DNA methylation was measured using the Illumina Infinium MethylationEPIC v1.0 BeadChip (n = 463) and the Illumina Infinium MethylationEPIC v2.0 BeadChip (n = 833). Raw intensity data (IDAT files) from the Illumina Infinium HumanMethylationEPIC BeadChip were processed using the SeSAMe pipeline (version 1.20.0) [Zhou et al., 2018]. Preprocessing was performed using the *openSesame()* function, which implements an integrated workflow including background correction, dye-bias correction, normalization, and masking of unreliable probes. Specifically, background correction was performed using the normal-exponential out-of-band (noob) method, which estimates background fluorescence from out-of-band probe intensities and subtracts it on a per-sample basis. Dye-bias correction was applied to adjust for systematic differences between the red and green color channels. The pipeline further performs signal normalization to reduce technical variation while preserving biological differences. Beta values, representing the proportion of methylation at each CpG site (ratio of methylated signal to total signal), were extracted for downstream statistical analyses. Within the SeSAMe workflow, “unreliable probes” refer to probes whose measured signal is not considered statistically distinguishable from background noise or that fail internal quality metrics. The masking of unreliable probes in SeSAMe is primarily based on detection p-values derived from signal-to-noise modeling. More specifically, SeSAMe evaluates probe performance using its pOOBAH (p-value with Out-Of-Band Array Hybridization) method. This approach estimates background signal distribution using out-of-band intensities and calculates a detection p-value for each probe in each sample. Probes with detection p-values exceeding the default threshold (p-value > 0.05) are considered not reliably detected, meaning their signal cannot be confidently distinguished from background fluorescence. These probe measurements are masked prior to downstream analysis by setting them to missing. In addition to poor detection, probes may also be masked if they exhibit extremely low signal intensity consistent with technical failure, show evidence of poor hybridization performance or map ambiguously or are prone to cross-hybridization (as defined in SeSAMe’s internal annotation resources) by masking rather than retaining these unreliable measurements, the pipeline reduces the inclusion of technical artifacts that could otherwise introduce bias or inflate false-positive findings. Only probes passing SeSAMe’s internal quality filters were retained for downstream analyses.

# **Supplementary results**

| **Supplementary Table 1.** Characteristics of the cytokine data. | | | | | | | | |
| --- | --- | --- | --- | --- | --- | --- | --- | --- |
| Inflammatory markers (49)* | Valid measurement | Missing / Outside standard range | 2011 sample size | 2018 sample size | Mean | Standard deviation | min | max |
| C-reactive protein | 2,193 | 38 | 1321 | 1115 | 1.85 | 3.80 | 0.07 | 98.34 |
| Cytokines, chemokines, and growth factors (48) | |  |  |  |  |  |  |  |
| Basic fibroblast growth factor | 2,187 | 44 | 1317 | 1112 | 73.25 | 36.53 | 7.16 | 869.10 |
| Beta-nerve growth factor | 2,115 | 116 | 1288 | 1070 | 1.52 | 1.13 | 0.06 | 34.92 |
| Cutaneous T-cell–attracting chemokine (CCL27) | 2,189 | 42 | 1318 | 1113 | 848.25 | 247.17 | 100.56 | 2,291.85 |
| Eotaxin (CCL11) | 2,181 | 50 | 1315 | 1109 | 153.62 | 655.73 | 11.99 | 29,287.64 |
| Granulocyte colony-stimulating factor | 2,188 | 43 | 1317 | 1112 | 151.22 | 338.14 | 24.27 | 15,825.00 |
| Granulocyte-macrophage colony-stimulating factor | 182 | 2,049 | 111 | 96 | 105.53 | 256.52 | 0.15 | 1,827.48 |
| Growth-regulated oncogene-α (CXCL1) | 2,171 | 60 | 1306 | 1100 | 91.14 | 47.62 | 4.19 | 673.69 |
| Hepatocyte growth factor | 2,189 | 42 | 1318 | 1113 | 551.03 | 208.67 | 27.11 | 1,891.76 |
| Interferon gamma | 2,189 | 42 | 1318 | 1113 | 285.00 | 199.21 | 36.53 | 6,271.36 |
| Interferon gamma–induced protein 10 (CXCL10) | 2,189 | 42 | 1318 | 1113 | 723.33 | 533.48 | 150.94 | 8,998.69 |
| Interleukin-1 receptor antagonist | 2,189 | 42 | 1318 | 1113 | 642.70 | 11,147.19 | 10.85 | 506,430.45 |
| Interleukin-10 | 2,186 | 45 | 1317 | 1111 | 28.76 | 129.34 | 0.63 | 3,547.46 |
| Interleukin-12 p40 subunit | 10 | 2,221 | 5 | 6 | 1,807.17 | 3,398.64 | 17.73 | 11,327.35 |
| Interleukin-12 p70 | 2,189 | 42 | 1318 | 1113 | 90.98 | 270.48 | 0.22 | 8,339.07 |
| Interleukin-13 | 2,189 | 42 | 1318 | 1113 | 21.76 | 52.40 | 1.40 | 1,479.70 |
| Interleukin-15 | 634 | 1,597 | 381 | 309 | 24.00 | 128.12 | 0.10 | 2,062.70 |
| Interleukin-16 | 2,013 | 218 | 1194 | 1030 | 85.70 | 62.45 | 0.46 | 1,813.30 |
| Interleukin-17 | 2,189 | 42 | 1318 | 1113 | 284.38 | 115.81 | 4.07 | 2,229.17 |
| Interleukin-18 | 2,189 | 42 | 1318 | 1113 | 73.07 | 38.25 | 7.13 | 647.38 |
| Interleukin-1α | 118 | 2,113 | 78 | 60 | 11.17 | 71.59 | 0.00 | 775.24 |
| Interleukin-1β | 2,188 | 43 | 1317 | 1112 | 5.17 | 2.21 | 0.16 | 48.34 |
| Interleukin-2 | 2,186 | 45 | 1317 | 1112 | 26.11 | 99.76 | 0.60 | 2,452.10 |
| Interleukin-2 receptor α (CD25) | 2,182 | 49 | 1315 | 1110 | 86.06 | 57.40 | 0.20 | 1,345.00 |
| Interleukin-3 | 11 | 2,220 | 4 | 5 | 658.47 | 544.42 | 24.90 | 1,641.80 |
| Interleukin-4 | 2,189 | 42 | 1318 | 1113 | 11.49 | 2.14 | 0.99 | 47.75 |
| Interleukin-5 | 2,187 | 44 | 1317 | 1112 | 6.68 | 11.47 | 1.85 | 524.30 |
| Interleukin-6 | 2,188 | 43 | 1317 | 1112 | 15.90 | 60.63 | 0.51 | 2,048.73 |
| Interleukin-7 | 2,189 | 42 | 1318 | 1113 | 24.23 | 44.98 | 0.99 | 983.85 |
| Interleukin-8 (CXCL8) | 2,189 | 42 | 1318 | 1113 | 32.71 | 9.04 | 3.95 | 154.54 |
| Interleukin-9 | 2,186 | 45 | 1316 | 1111 | 324.17 | 5,099.46 | 5.78 | 176,521.00 |
| Leukemia inhibitory factor | 16 | 2,215 | 11 | 8 | 787.2 | 1,976.92 | 1.55 | 8,033.91 |
| Macrophage colony-stimulating factor | 950 | 1,281 | 576 | 478 | 4.30 | 10.78 | 0.01 | 232.85 |
| Macrophage inflammatory protein-1α (CCL3) | 2,189 | 42 | 1318 | 1113 | 13.42 | 15.58 | 2.61 | 716.60 |
| Macrophage inflammatory protein-1β (CCL4) | 2,189 | 42 | 1318 | 1113 | 91.24 | 69.20 | 21.54 | 2,992.00 |
| Macrophage migration inhibitory factor | 2,187 | 44 | 1317 | 1112 | 181.49 | 136.49 | 4.28 | 2,365.60 |
| Monocyte chemoattractant protein-1 (CCL2) | 2,189 | 42 | 1318 | 1113 | 35.57 | 22.14 | 2.49 | 675.72 |
| Monocyte chemoattractant protein-3 (CCL7) | 278 | 1,953 | 175 | 131 | 26.07 | 67.73 | 0.05 | 733.41 |
| Monokine induced by IFN-γ (CXCL9) | 2,189 | 42 | 1318 | 1113 | 574.31 | 535.05 | 105.19 | 7,594.57 |
| Platelet-derived growth factor-BB | 2,189 | 42 | 1318 | 1113 | 8,948.074 | 3,130.70 | 252.85 | 32,467.34 |
| Stem cell factor (KIT ligand) | 2,188 | 43 | 1317 | 1112 | 97.13 | 187.50 | 7.11 | 8,744.00 |
| Stem cell growth factor-β | 2,187 | 44 | 1316 | 1112 | 11,769.81 | 4,901.98 | 1,178.54 | 59,821.31 |
| Stromal cell-derived factor 1α (CXCL12) | 1,982 | 249 | 1180 | 998 | 72.78 | 34.03 | 5.91 | 655.66 |
| TNF-related apoptosis-inducing ligand | 2,182 | 49 | 1313 | 1112 | 149.83 | 116.34 | 13.41 | 1,961.15 |
| Tumor necrosis factor-α | 2,189 | 42 | 1318 | 1113 | 64.49 | 217.52 | 0.29 | 8,434.11 |
| Tumor necrosis factor-β (lymphotoxin) | 128 | 2,103 | 79 | 67 | 17.93 | 51.434 | 0.01 | 469.91 |
| Vascular endothelial growth factor | 2,189 | 42 | 1318 | 1113 | 82.39 | 47.27 | 2.62 | 585.27 |
| CCL5 | 0 | 2200 | 0 | 0 | - | - | - | - |
| Interferon-α 2 | 0 | 2200 | 0 | 0 | - | - | - | - |
| This table summarizes descriptive statistics and sample availability for the 47 inflammatory markers measured at baseline and at two follow‑up waves. “Within standard range” and “Outside standard range” refer to the number of biomarker values falling inside or outside the manufacturer‑specified assay detection or reference ranges at baseline. “2011 sample size” and “2018 sample size” denote the number of participants with valid cytokine measurements at each respective follow‑up. Mean, standard deviation, minimum, and maximum values describe the distribution of each cytokine at baseline after quality‑control procedures. Rows highlighted in red indicate markers excluded from further analyses due to insufficient sample size.  *48 cytokines and C-reactive protein | | | | | | | | |

| **Supplementary Table 2.** Longitudinal associations between inflammatory biomarkers and DunedinPACE | | | | | | | | | | | | | | | | | |  |
| --- | --- | --- | --- | --- | --- | --- | --- | --- | --- | --- | --- | --- | --- | --- | --- | --- | --- | --- |
| **Inflammatory markers** | **DunedinPACE, 2011** | | | | | | | | | **DunedinPACE, 2018** | | | | | | | |  |
|  | **Model 1** | | | **Model 2** | | | **Model 3** | | | **Model 1** | | | **Model 2** | | | **Model 3** | | |
|  | **β ± Std. Error** | **P-value** | **Adj.P-value** | **β ± Std. Error** | **P-value** | **Adj. P-value** | **β ± Std. Error** | **P-value** | **Adj. P-value** | **β ± Std. Error** | **P-value** | **Adj.P-value** | **β ± Std. Error** | **P-value** | **Adj. P-value** | **β ± Std. Error** | **P-value** | **Adj. P-value** |
| Combined score ^##^ | 0.017 ± 0.001 | 6.32E-34 | 2.46E-32 | 0.01 ± 0.001 | 1.67826E-13 | 6.54523E-12 | 0.01 ± 0.001 | 1.1804E-13 | 4.60356E-12 | 0.017 ± 0.002 | 3.05E-21 | 1.19E-19 | 0.011 ± 0.002 | 1.26E-08 | 4.93E-07 | 0.011 ± 0.002 | 9.15E-08 | 3.57E-06 |
| **Pro-inflammatory** |  |  |  |  |  |  |  |  |  |  |  |  |  |  |  |  |  |  |
| log(CRP) | 0.022 ± 0.002 | 1.08E-23 | 4.12E-22 | 0.011 ± 0.002 | 3.58E-07 | 6.80E-06 | 0.011 ± 0.002 | 4.24E-07 | 5.37E-06 | 0.019 ± 0.003 | 2.17E-11 | 2.82E-10 | 0.008 ± 0.003 | 0.010 | 0.022 | 0.006 ± 0.003 | 0.044 | 0.065 |
| log(Eotaxin) | 0.013 ± 0.005 | 0.012 | 0.028 | 0.012 ± 0.005 | 0.007 | 0.025 | 0.012 ± 0.004 | 0.006 | 0.019 | 0.018 ± 0.007 | 0.012 | 0.026 | 0.028 ± 0.007 | 7.25E-05 | 0.001 | 0.028 ± 0.007 | 5.77E-05 | 3.21E-04 |
| log(FGFBasic) | 0.019 ± 0.008 | 0.017 | 0.035 | 0.014 ± 0.007 | 0.056 | 0.119 | 0.014 ± 0.007 | 0.038 | 0.084 | 0.03 ± 0.01 | 0.003 | 0.011 | 0.031 ± 0.01 | 0.003 | 0.009 | 0.034 ± 0.01 | 0.001 | 0.003 |
| log(GCSF) | 0.024 ± 0.008 | 0.004 | 0.011 | 0.022 ± 0.007 | 0.003 | 0.017 | 0.022 ± 0.007 | 0.002 | 0.011 | 0.041 ± 0.012 | 4.93E-04 | 0.002 | 0.045 ± 0.012 | 1.31E-04 | 0.001 | 0.047 ± 0.011 | 4.32E-05 | 2.81E-04 |
| log(IL12p70) | 0.017 ± 0.004 | 7.15E-05 | 3.40E-04 | 0.01 ± 0.004 | 0.010 | 0.032 | 0.009 ± 0.004 | 0.021 | 0.056 | 0.014 ± 0.005 | 0.010 | 0.024 | 0.007 ± 0.006 | 0.235 | 0.316 | 0.008 ± 0.006 | 0.174 | 0.219 |
| log(IL17) | 0.001 ± 0.008 | 0.911 | 0.911 | 0.003 ± 0.007 | 0.726 | 0.746 | 0.004 ± 0.007 | 0.597 | 0.731 | 0.025 ± 0.01 | 0.016 | 0.033 | 0.028 ± 0.011 | 0.008 | 0.021 | 0.031 ± 0.01 | 0.003 | 0.009 |
| log(IL18) | 0.039 ± 0.006 | 1.31E-11 | 1.66E-10 | 0.025 ± 0.005 | 1.37E-06 | 1.74E-05 | 0.026 ± 0.005 | 3.61E-07 | 5.37E-06 | 0.048 ± 0.007 | 1.03E-10 | 1.00E-09 | 0.031 ± 0.008 | 4.20E-05 | 4.10E-04 | 0.031 ± 0.007 | 3.90E-05 | 2.81E-04 |
| log(IL6) | 0.014 ± 0.006 | 0.016 | 0.034 | 0.01 ± 0.005 | 0.056 | 0.119 | 0.011 ± 0.005 | 0.026 | 0.063 | 0.001 ± 0.007 | 0.887 | 0.887 | 0.008 ± 0.008 | 0.302 | 0.357 | 0.011 ± 0.007 | 0.150 | 0.202 |
| log(MIP1b) | 0.012 ± 0.008 | 0.125 | 0.176 | 0.004 ± 0.007 | 0.575 | 0.649 | 0.003 ± 0.007 | 0.627 | 0.739 | 0.007 ± 0.01 | 0.498 | 0.539 | 0.007 ± 0.01 | 0.453 | 0.520 | 0.009 ± 0.01 | 0.355 | 0.396 |
| log(MCP1) | 0.014 ± 0.007 | 0.050 | 0.082 | 0.01 ± 0.007 | 0.131 | 0.205 | 0.01 ± 0.006 | 0.111 | 0.175 | 0.026 ± 0.009 | 0.003 | 0.011 | 0.03 ± 0.009 | 0.002 | 0.007 | 0.027 ± 0.009 | 0.003 | 0.009 |
| PDGFbb | 0 ± 0 | 0.159 | 0.195 | 0 ± 0 | 0.614 | 0.667 | 0 ± 0 | 0.661 | 0.739 | 0 ± 0 | 0.067 | 0.093 | 0 ± 0 | 0.030 | 0.051 | 0 ± 0 | 0.028 | 0.045 |
| log(SCF) | -0.007 ± 0.007 | 0.300 | 0.335 | -0.004 ± 0.006 | 0.513 | 0.609 | -0.004 ± 0.006 | 0.480 | 0.608 | 0.01 ± 0.009 | 0.291 | 0.334 | 0.006 ± 0.009 | 0.506 | 0.548 | 0.003 ± 0.009 | 0.702 | 0.740 |
| log(SDF1a) | -0.002 ± 0.006 | 0.688 | 0.707 | 0.004 ± 0.005 | 0.455 | 0.565 | 0 ± 0.005 | 0.972 | 0.972 | 0.01 ± 0.007 | 0.134 | 0.174 | 0.017 ± 0.007 | 0.019 | 0.035 | 0.017 ± 0.007 | 0.016 | 0.030 |
| log(TRAIL) | 0.008 ± 0.005 | 0.140 | 0.186 | -0.003 ± 0.005 | 0.581 | 0.649 | 0 ± 0.005 | 0.968 | 0.972 | 0.015 ± 0.007 | 0.022 | 0.038 | 0.005 ± 0.007 | 0.493 | 0.548 | 0.007 ± 0.007 | 0.257 | 0.295 |
| log(VEGF) | 0.02 ± 0.005 | 2.30E-05 | 1.45E-04 | 0.01 ± 0.004 | 0.028 | 0.071 | 0.007 ± 0.004 | 0.090 | 0.155 | 0.021 ± 0.006 | 0.001 | 0.003 | 0.008 ± 0.006 | 0.222 | 0.309 | 0.008 ± 0.006 | 0.187 | 0.221 |
| log(IL2) | 0.008 ± 0.006 | 0.142 | 0.186 | 0.008 ± 0.005 | 0.105 | 0.191 | 0.01 ± 0.005 | 0.041 | 0.087 | 0.003 ± 0.007 | 0.678 | 0.714 | 0.012 ± 0.007 | 0.098 | 0.147 | 0.016 ± 0.007 | 0.022 | 0.039 |
| log(IL9) | 0.007 ± 0.003 | 0.048 | 0.082 | 0.005 ± 0.003 | 0.127 | 0.205 | 0.005 ± 0.003 | 0.101 | 0.167 | 0.01 ± 0.004 | 0.021 | 0.038 | 0.005 ± 0.004 | 0.252 | 0.328 | 0.006 ± 0.004 | 0.170 | 0.219 |
| log(IL16) | 0.005 ± 0.004 | 0.147 | 0.186 | 0.002 ± 0.003 | 0.461 | 0.565 | 0.002 ± 0.003 | 0.641 | 0.739 | 0.006 ± 0.005 | 0.239 | 0.282 | 0.002 ± 0.005 | 0.726 | 0.745 | 0.001 ± 0.005 | 0.891 | 0.914 |
| log(CTACK) | -0.038 ± 0.008 | 7.79E-06 | 5.92E-05 | -0.002 ± 0.008 | 0.769 | 0.769 | -0.003 ± 0.008 | 0.713 | 0.774 | -0.029 ± 0.011 | 0.010 | 0.024 | -0.002 ± 0.012 | 0.868 | 0.868 | 0.001 ± 0.012 | 0.918 | 0.918 |
| log(GROa) | 0.008 ± 0.005 | 0.099 | 0.145 | 0.006 ± 0.005 | 0.152 | 0.223 | 0.006 ± 0.004 | 0.187 | 0.263 | 0.009 ± 0.006 | 0.146 | 0.184 | 0.017 ± 0.006 | 0.008 | 0.021 | 0.016 ± 0.006 | 0.013 | 0.027 |
| log(MIF) | 0.008 ± 0.004 | 0.054 | 0.086 | 0.008 ± 0.004 | 0.048 | 0.113 | 0.006 ± 0.004 | 0.084 | 0.153 | 0.013 ± 0.005 | 0.017 | 0.033 | 0.006 ± 0.005 | 0.288 | 0.351 | 0.007 ± 0.005 | 0.182 | 0.221 |
| log(MIG) | 0.002 ± 0.005 | 0.682 | 0.707 | 0.007 ± 0.004 | 0.121 | 0.205 | 0.007 ± 0.004 | 0.115 | 0.175 | 0.021 ± 0.006 | 4.58E-04 | 0.002 | 0.018 ± 0.006 | 0.004 | 0.011 | 0.016 ± 0.006 | 0.007 | 0.016 |
| log(bNGF) | 0.006 ± 0.005 | 0.207 | 0.243 | 0.007 ± 0.005 | 0.135 | 0.205 | 0.008 ± 0.005 | 0.072 | 0.138 | 0.012 ± 0.007 | 0.066 | 0.093 | 0.016 ± 0.007 | 0.020 | 0.036 | 0.018 ± 0.007 | 0.007 | 0.016 |
| log(SCGFb) | -0.008 ± 0.006 | 0.211 | 0.243 | 0.003 ± 0.006 | 0.671 | 0.708 | 0.002 ± 0.006 | 0.781 | 0.824 | -0.001 ± 0.008 | 0.869 | 0.887 | 0.005 ± 0.009 | 0.578 | 0.610 | 0.004 ± 0.008 | 0.649 | 0.703 |
| log(TNFa) | 0.013 ± 0.005 | 0.014 | 0.031 | 0.012 ± 0.005 | 0.011 | 0.032 | 0.014 ± 0.005 | 0.003 | 0.014 | 0.009 ± 0.007 | 0.180 | 0.220 | 0.023 ± 0.007 | 0.002 | 0.007 | 0.026 ± 0.007 | 3.41E-04 | 0.002 |
| log(IL5) | 0.028 ± 0.008 | 1.82E-04 | 0.001 | 0.022 ± 0.007 | 0.001 | 0.010 | 0.023 ± 0.007 | 5.00E-04 | 0.004 | 0.026 ± 0.01 | 0.012 | 0.026 | 0.024 ± 0.01 | 0.018 | 0.035 | 0.027 ± 0.01 | 0.006 | 0.015 |
| log(IL7) | 0.018 ± 0.006 | 0.004 | 0.010 | 0.014 ± 0.006 | 0.009 | 0.031 | 0.014 ± 0.005 | 0.008 | 0.026 | 0.021 ± 0.008 | 0.008 | 0.023 | 0.025 ± 0.008 | 0.002 | 0.009 | 0.026 ± 0.008 | 0.001 | 0.004 |
| log(IL8) | 0.036 ± 0.011 | 0.001 | 0.004 | 0.028 ± 0.01 | 0.005 | 0.022 | 0.031 ± 0.01 | 0.002 | 0.011 | 0.056 ± 0.014 | 6.97E-05 | 0.001 | 0.055 ± 0.014 | 0.000 | 0.001 | 0.059 ± 0.014 | 1.60E-05 | 1.56E-04 |
| log(MIP1a) | 0.021 ± 0.009 | 0.020 | 0.038 | 0.019 ± 0.008 | 0.020 | 0.055 | 0.021 ± 0.008 | 0.009 | 0.026 | 0.043 ± 0.013 | 0.001 | 0.004 | 0.044 ± 0.013 | 0.001 | 0.004 | 0.045 ± 0.013 | 3.55E-04 | 0.002 |
| log(IP10) | 0.003 ± 0.005 | 0.514 | 0.559 | 0.005 ± 0.005 | 0.262 | 0.344 | 0.004 ± 0.005 | 0.325 | 0.425 | 0.015 ± 0.006 | 0.022 | 0.038 | 0.017 ± 0.007 | 0.010 | 0.022 | 0.016 ± 0.006 | 0.016 | 0.030 |
| IL1b07 | 0.005 ± 0.001 | 5.89E-05 | 3.20E-04 | 0.004 ± 0.001 | 2.85E-04 | 0.003 | 0.004 ± 0.001 | 8.78E-05 | 0.001 | 0.004 ± 0.001 | 0.009 | 0.024 | 0.006 ± 0.001 | 3.14E-05 | 4.08E-04 | 0.006 ± 0.001 | 5.69E-06 | 7.40E-05 |
| log(IL2ra) | 0.022 ± 0.004 | 5.15E-07 | 4.89E-06 | 0.012 ± 0.004 | 0.003 | 0.017 | 0.012 ± 0.004 | 0.002 | 0.011 | 0.023 ± 0.006 | 1.19E-04 | 0.001 | 0.016 ± 0.006 | 0.010 | 0.022 | 0.017 ± 0.006 | 0.006 | 0.015 |
| log(IFNg) | 0.022 ± 0.008 | 0.006 | 0.015 | 0.009 ± 0.007 | 0.220 | 0.298 | 0.008 ± 0.007 | 0.256 | 0.347 | 0.02 ± 0.01 | 0.048 | 0.075 | 0.019 ± 0.011 | 0.076 | 0.123 | 0.022 ± 0.01 | 0.034 | 0.053 |
| **Anti-inflammatory** |  |  |  |  |  |  |  |  |  |  |  |  |  |  |  |  |  |  |
| log(HGF) | 0.057 ± 0.006 | 5.23E-19 | 9.93E-18 | 0.035 ± 0.006 | 7.21E-09 | 2.74E-07 | 0.034 ± 0.006 | 1.08E-08 | 4.10E-07 | 0.075 ± 0.009 | 1.11E-17 | 2.17E-16 | 0.048 ± 0.009 | 2.40E-07 | 4.67E-06 | 0.045 ± 0.009 | 5.89E-07 | 1.15E-05 |
| log(IL1ra) | 0.01 ± 0.005 | 0.028 | 0.050 | 0.007 ± 0.004 | 0.065 | 0.123 | 0.009 ± 0.004 | 0.026 | 0.063 | 0.006 ± 0.006 | 0.324 | 0.361 | 0.01 ± 0.006 | 0.086 | 0.135 | 0.013 ± 0.006 | 0.025 | 0.042 |
| log(IL10) | 0.015 ± 0.004 | 1.88E-04 | 0.001 | 0.01 ± 0.004 | 0.005 | 0.022 | 0.01 ± 0.004 | 0.005 | 0.019 | 0.01 ± 0.005 | 0.055 | 0.082 | 0.006 ± 0.006 | 0.275 | 0.346 | 0.008 ± 0.005 | 0.140 | 0.195 |
| log(IL4) | 0.022 ± 0.013 | 0.095 | 0.145 | 0.016 ± 0.012 | 0.175 | 0.246 | 0.017 ± 0.011 | 0.136 | 0.199 | 0.028 ± 0.016 | 0.087 | 0.117 | 0.041 ± 0.017 | 0.015 | 0.031 | 0.046 ± 0.016 | 0.005 | 0.014 |
| log(IL13) | 0.017 ± 0.005 | 0.002 | 0.006 | 0.009 ± 0.005 | 0.061 | 0.123 | 0.009 ± 0.005 | 0.050 | 0.101 | 0.014 ± 0.007 | 0.036 | 0.059 | 0.01 ± 0.007 | 0.178 | 0.257 | 0.011 ± 0.007 | 0.115 | 0.166 |
| Associations between inflammatory biomarkers (38, measured in 2007) and DunedinPACE (measured in 2011, and 2018) were determined using three linear regression models adjusting for different covariate sets ^#^. Regression coefficients (β), standard errors, P-values, and false discovery rate adjusted (FDR) P-values are reported.  *Inflammatory biomarkers highlighted in green are associated robustly in both 2011 and 2018 follow-ups. P-values highlighted in yellow are statistically significant. ^#^ Three models were considered with model 1 adjusting only for chronological age and sex, model 2 adjusting additionally for BMI, smoking status, socioeconomic status, alcohol use and physical activity (MET), and model 3 adjusting for all previously mentioned covariates and cell proportions. All 2018 analyses were additionally adjusted for a technical covariates “batches”, representing the two batches of DNA methylation profiling and different versions of EPIC arrays. ^##^ **Combined score:** The combined score estimating systemic inflammation is a sum of five log-transformed inflammatory biomarkers (CRP, Eotaxin, IL-18, IL2-r𝛼, HGF). | | | | | | | | | | | | | | | | | | |

| **Supplementary Table 3.** Longitudinal associations between inflammatory biomarkers and PCGrimAgeDev | | | | | | | | | | | | | | | | | | |
| --- | --- | --- | --- | --- | --- | --- | --- | --- | --- | --- | --- | --- | --- | --- | --- | --- | --- | --- |
| **Inflammatory markers** | **PCGrimAgeDev, 2011** | | | | | | | | | **PCGrimAgeDev, 2018** | | | | | | | | |
|  | **Model 1** | | | **Model 2** | | | **Model 3** | | | **Model 1** | | | **Model 2** | | | **Model 3** | | |
|  | **β ± Std. Error** | **P-value** | **Adj. P-value** | **β ± Std. Error** | **P-value** | **Adj. P-value** | **β ± Std. Error** | **P-value** | **Adj. P-value** | **β ± Std. Error** | **P-value** | **Adj. P-value** | **β ± Std. Error** | **P-value** | **Adj. P-value** | **β ± Std. Error** | **P-value** | **Adj. P-value** |
| Combined score^##^ | 0.355 ± 0.046 | 4.3154E-14 | 1.683E-12 | 0.202 ± 0.041 | 9.122E-07 | 3.5576E-05 | 0.187 ± 0.036 | 1.9339E-07 | 7.5423E-06 | 0.306 ± 0.054 | 2.16E-08 | 8.44E-07 | 0.19 ± 0.054 | 4.86E-04 | 0.019 | 0.13 ± 0.049 | 0.008 | 0.286 |
| **Pro-inflammatory** | |  |  |  |  |  |  |  |  |  |  |  |  |  |  |  |  |  |
| log(CRP) | 0.347 ± 0.075 | 4.66E-06 | 7.83E-05 | 0.177 ± 0.068 | 0.009 | 0.058 | 0.169 ± 0.059 | 0.005 | 0.035 | 0.308 ± 0.086 | 3.40E-04 | 0.003 | 0.207 ± 0.087 | 0.017 | 0.223 | 0.142 ± 0.078 | 0.069 | 0.336 |
| log(Eotaxin) | 0.708 ± 0.172 | 3.94E-05 | 2.99E-04 | 0.445 ± 0.138 | 0.001 | 0.024 | 0.418 ± 0.121 | 0.001 | 0.011 | 0.375 ± 0.206 | 0.069 | 0.201 | 0.354 ± 0.193 | 0.068 | 0.376 | 0.284 ± 0.172 | 0.100 | 0.434 |
| log(FGFBasic) | 0.499 ± 0.271 | 0.065 | 0.146 | 0.211 ± 0.215 | 0.328 | 0.622 | 0.311 ± 0.189 | 0.100 | 0.293 | 0.202 ± 0.301 | 0.502 | 0.789 | 0.135 ± 0.279 | 0.628 | 0.972 | 0.246 ± 0.248 | 0.321 | 0.883 |
| log(GCSF) | 0.622 ± 0.283 | 0.028 | 0.083 | 0.377 ± 0.227 | 0.098 | 0.337 | 0.414 ± 0.2 | 0.039 | 0.183 | 0.63 ± 0.35 | 0.072 | 0.201 | 0.398 ± 0.317 | 0.210 | 0.817 | 0.378 ± 0.282 | 0.181 | 0.626 |
| log(IL12p70) | 0.495 ± 0.143 | 0.001 | 0.003 | 0.263 ± 0.116 | 0.023 | 0.090 | 0.205 ± 0.102 | 0.043 | 0.183 | 0.308 ± 0.162 | 0.058 | 0.201 | 0.072 ± 0.153 | 0.638 | 0.972 | 0.044 ± 0.136 | 0.747 | 0.957 |
| log(IL17) | -0.116 ± 0.278 | 0.677 | 0.780 | -0.151 ± 0.22 | 0.493 | 0.747 | -0.052 ± 0.193 | 0.789 | 0.937 | 0.168 ± 0.303 | 0.580 | 0.838 | 0.091 ± 0.286 | 0.749 | 0.972 | 0.156 ± 0.255 | 0.541 | 0.957 |
| log(IL18) | 0.877 ± 0.193 | 6.18E-06 | 7.83E-05 | 0.566 ± 0.158 | 3.63E-04 | 0.014 | 0.544 ± 0.139 | 9.47E-05 | 0.004 | 0.943 ± 0.219 | 1.76E-05 | 2.29E-04 | 0.458 ± 0.207 | 0.027 | 0.268 | 0.365 ± 0.185 | 0.049 | 0.286 |
| log(IL6) | 0.019 ± 0.198 | 0.925 | 0.925 | -0.085 ± 0.157 | 0.590 | 0.747 | -0.013 ± 0.138 | 0.927 | 0.967 | 0.018 ± 0.215 | 0.934 | 0.934 | 0.028 ± 0.203 | 0.892 | 0.972 | 0.052 ± 0.181 | 0.774 | 0.957 |
| log(MIP1b) | 0.132 ± 0.264 | 0.616 | 0.767 | 0.128 ± 0.214 | 0.549 | 0.747 | 0.079 ± 0.188 | 0.672 | 0.851 | 0.03 ± 0.289 | 0.916 | 0.934 | 0.049 ± 0.267 | 0.854 | 0.972 | 0.027 ± 0.238 | 0.909 | 0.957 |
| log(MCP1) | 0.838 ± 0.243 | 0.001 | 0.003 | 0.564 ± 0.198 | 0.004 | 0.034 | 0.573 ± 0.173 | 0.001 | 0.012 | 0.702 ± 0.26 | 0.007 | 0.046 | 0.486 ± 0.252 | 0.054 | 0.350 | 0.499 ± 0.225 | 0.027 | 0.286 |
| PDGFbb | 0 ± 0 | 0.748 | 0.836 | 0 ± 0 | 0.956 | 0.956 | 0 ± 0 | 0.909 | 0.967 | 0 ± 0 | 0.871 | 0.934 | 0 ± 0 | 0.580 | 0.972 | 0 ± 0 | 0.707 | 0.957 |
| log(SCF) | -0.222 ± 0.232 | 0.339 | 0.460 | 0.114 ± 0.188 | 0.545 | 0.747 | 0.032 ± 0.166 | 0.846 | 0.967 | -0.063 ± 0.27 | 0.815 | 0.934 | -0.049 ± 0.25 | 0.846 | 0.972 | -0.189 ± 0.223 | 0.396 | 0.883 |
| log(SDF1a) | 0.374 ± 0.192 | 0.051 | 0.130 | 0.398 ± 0.156 | 0.011 | 0.059 | 0.185 ± 0.137 | 0.176 | 0.393 | -0.039 ± 0.204 | 0.850 | 0.934 | 0.021 ± 0.198 | 0.916 | 0.972 | 0.031 ± 0.176 | 0.861 | 0.957 |
| log(TRAIL) | 0.082 ± 0.179 | 0.646 | 0.767 | -0.085 ± 0.149 | 0.568 | 0.747 | 0.095 ± 0.131 | 0.468 | 0.741 | -0.023 ± 0.193 | 0.907 | 0.934 | -0.106 ± 0.182 | 0.559 | 0.972 | 0.009 ± 0.162 | 0.957 | 0.957 |
| log(VEGF) | 0.642 ± 0.16 | 6.12E-05 | 3.87E-04 | 0.31 ± 0.132 | 0.019 | 0.089 | 0.197 ± 0.116 | 0.089 | 0.293 | 0.525 ± 0.181 | 0.004 | 0.029 | 0.168 ± 0.175 | 0.337 | 0.972 | 0.129 ± 0.156 | 0.408 | 0.883 |
| log(IL2) | -0.03 ± 0.188 | 0.872 | 0.895 | -0.063 ± 0.149 | 0.673 | 0.809 | 0.047 ± 0.131 | 0.721 | 0.884 | -0.035 ± 0.206 | 0.864 | 0.934 | 0.047 ± 0.19 | 0.805 | 0.972 | 0.066 ± 0.17 | 0.698 | 0.957 |
| log(IL9) | 0.157 ± 0.113 | 0.166 | 0.274 | 0.109 ± 0.09 | 0.226 | 0.536 | 0.102 ± 0.079 | 0.199 | 0.419 | 0.313 ± 0.127 | 0.014 | 0.069 | 0.113 ± 0.113 | 0.320 | 0.972 | 0.113 ± 0.101 | 0.262 | 0.787 |
| log(IL16) | 0.239 ± 0.127 | 0.060 | 0.143 | 0.125 ± 0.103 | 0.223 | 0.536 | 0.089 ± 0.091 | 0.324 | 0.585 | 0.052 ± 0.139 | 0.707 | 0.934 | -0.077 ± 0.136 | 0.569 | 0.972 | -0.161 ± 0.122 | 0.186 | 0.626 |
| log(CTACK) | -0.425 ± 0.287 | 0.139 | 0.251 | 0.157 ± 0.244 | 0.519 | 0.747 | 0.123 ± 0.214 | 0.565 | 0.792 | -0.475 ± 0.331 | 0.152 | 0.330 | -0.066 ± 0.322 | 0.838 | 0.972 | -0.124 ± 0.288 | 0.667 | 0.957 |
| log(GROa) | 0.079 ± 0.169 | 0.641 | 0.767 | 0.075 ± 0.137 | 0.587 | 0.747 | 0.009 ± 0.121 | 0.943 | 0.967 | -0.175 ± 0.189 | 0.353 | 0.651 | 0.083 ± 0.175 | 0.635 | 0.972 | 0.035 ± 0.156 | 0.823 | 0.957 |
| log(MIF) | 0.338 ± 0.142 | 0.017 | 0.060 | 0.342 ± 0.115 | 0.003 | 0.029 | 0.274 ± 0.101 | 0.007 | 0.044 | 0.298 ± 0.16 | 0.063 | 0.201 | 0.013 ± 0.147 | 0.931 | 0.972 | 0.027 ± 0.131 | 0.839 | 0.957 |
| log(MIG) | -0.264 ± 0.159 | 0.098 | 0.203 | 0.014 ± 0.129 | 0.911 | 0.956 | -0.005 ± 0.113 | 0.967 | 0.967 | 0.218 ± 0.176 | 0.214 | 0.439 | 0.215 ± 0.163 | 0.186 | 0.807 | 0.051 ± 0.145 | 0.727 | 0.957 |
| log(bNGF) | -0.354 ± 0.167 | 0.034 | 0.093 | -0.149 ± 0.142 | 0.296 | 0.610 | -0.071 ± 0.125 | 0.569 | 0.792 | -0.301 ± 0.194 | 0.121 | 0.313 | -0.109 ± 0.182 | 0.551 | 0.972 | -0.144 ± 0.161 | 0.370 | 0.883 |
| log(SCGFb) | -0.615 ± 0.216 | 0.005 | 0.019 | -0.031 ± 0.18 | 0.863 | 0.937 | -0.096 ± 0.159 | 0.544 | 0.792 | -0.535 ± 0.24 | 0.026 | 0.103 | -0.274 ± 0.229 | 0.233 | 0.825 | -0.407 ± 0.205 | 0.047 | 0.286 |
| log(TNFa) | 0.112 ± 0.183 | 0.541 | 0.709 | -0.009 ± 0.147 | 0.954 | 0.956 | 0.112 ± 0.129 | 0.387 | 0.640 | -0.091 ± 0.208 | 0.661 | 0.921 | -0.008 ± 0.201 | 0.967 | 0.972 | 0.024 ± 0.179 | 0.893 | 0.957 |
| log(IL5) | 0.42 ± 0.256 | 0.101 | 0.203 | 0.215 ± 0.209 | 0.305 | 0.610 | 0.311 ± 0.184 | 0.092 | 0.293 | 0.273 ± 0.303 | 0.367 | 0.651 | 0.21 ± 0.269 | 0.435 | 0.972 | 0.313 ± 0.24 | 0.192 | 0.626 |
| log(IL7) | 0.285 ± 0.206 | 0.166 | 0.274 | 0.191 ± 0.168 | 0.255 | 0.569 | 0.179 ± 0.147 | 0.224 | 0.425 | 0.14 ± 0.23 | 0.541 | 0.812 | 0.038 ± 0.221 | 0.863 | 0.972 | 0.035 ± 0.197 | 0.861 | 0.957 |
| log(IL8) | 0.448 ± 0.379 | 0.238 | 0.353 | 0.2 ± 0.306 | 0.513 | 0.747 | 0.329 ± 0.268 | 0.220 | 0.425 | 0.953 ± 0.413 | 0.021 | 0.092 | 0.755 ± 0.379 | 0.046 | 0.350 | 0.687 ± 0.338 | 0.042 | 0.286 |
| log(MIP1a) | 0.355 ± 0.309 | 0.251 | 0.353 | 0.204 ± 0.246 | 0.407 | 0.737 | 0.341 ± 0.216 | 0.114 | 0.293 | 0.103 ± 0.383 | 0.789 | 0.934 | 0.126 ± 0.352 | 0.720 | 0.972 | 0.176 ± 0.313 | 0.574 | 0.957 |
| log(IP10) | -0.214 ± 0.166 | 0.199 | 0.316 | 0.186 ± 0.138 | 0.178 | 0.520 | 0.116 ± 0.123 | 0.345 | 0.596 | -0.175 ± 0.19 | 0.357 | 0.651 | 0.074 ± 0.177 | 0.677 | 0.972 | 0.071 ± 0.159 | 0.654 | 0.957 |
| IL1b | 0.057 ± 0.038 | 0.137 | 0.251 | 0.021 ± 0.03 | 0.492 | 0.747 | 0.036 ± 0.027 | 0.172 | 0.393 | 0.033 ± 0.044 | 0.450 | 0.763 | 0.065 ± 0.04 | 0.102 | 0.496 | 0.069 ± 0.035 | 0.051 | 0.286 |
| log(IL2ra) | 0.651 ± 0.149 | 0.000 | 0.000 | 0.273 ± 0.121 | 0.024 | 0.090 | 0.279 ± 0.106 | 0.009 | 0.046 | 0.444 ± 0.179 | 0.013 | 0.069 | 0.136 ± 0.172 | 0.428 | 0.972 | 0.073 ± 0.154 | 0.637 | 0.957 |
| log(IFNg) | 0.322 ± 0.276 | 0.244 | 0.353 | -0.091 ± 0.221 | 0.681 | 0.809 | -0.088 ± 0.194 | 0.651 | 0.851 | -0.033 ± 0.298 | 0.911 | 0.934 | -0.237 ± 0.285 | 0.405 | 0.972 | -0.044 ± 0.255 | 0.864 | 0.957 |
| **Anti-inflammatory** | |  |  |  |  |  |  |  |  |  |  |  |  |  |  |  |  |  |
| log(HGF) | 1.102 ± 0.218 | 5.23E-07 | 1.99E-05 | 0.569 ± 0.185 | 0.002 | 0.027 | 0.474 ± 0.162 | 0.004 | 0.033 | 1.306 ± 0.26 | 5.88E-07 | 1.15E-05 | 0.767 ± 0.249 | 0.002 | 0.042 | 0.502 ± 0.225 | 0.026 | 0.286 |
| log(IL1ra) | 0.033 ± 0.152 | 0.829 | 0.887 | -0.038 ± 0.12 | 0.750 | 0.863 | 0.058 ± 0.105 | 0.584 | 0.792 | 0.015 ± 0.167 | 0.929 | 0.934 | -0.006 ± 0.157 | 0.972 | 0.972 | 0.025 ± 0.14 | 0.858 | 0.957 |
| log(IL10) | 0.314 ± 0.14 | 0.025 | 0.078 | 0.163 ± 0.112 | 0.148 | 0.469 | 0.159 ± 0.099 | 0.108 | 0.293 | 0.234 ± 0.157 | 0.136 | 0.330 | 0.023 ± 0.148 | 0.878 | 0.972 | 0.008 ± 0.132 | 0.954 | 0.957 |
| log(IL4) | 0.09 ± 0.445 | 0.840 | 0.887 | -0.08 ± 0.357 | 0.823 | 0.919 | 0.032 ± 0.313 | 0.918 | 0.967 | 0.32 ± 0.481 | 0.506 | 0.789 | 0.408 ± 0.458 | 0.373 | 0.972 | 0.387 ± 0.408 | 0.343 | 0.883 |
| log(IL13) | 0.436 ± 0.182 | 0.017 | 0.060 | 0.18 ± 0.146 | 0.215 | 0.536 | 0.201 ± 0.128 | 0.116 | 0.293 | 0.293 ± 0.204 | 0.150 | 0.330 | 0.023 ± 0.193 | 0.907 | 0.972 | 0.054 ± 0.172 | 0.754 | 0.957 |
| Associations between inflammatory biomarkers (38, measured in 2007) and PCGrimAgeDev (measured in 2011, and 2018) were determined using three linear regression models adjusting for different covariate sets ^#^. Regression coefficients (β), standard errors, P-values, and false discovery rate adjusted (FDR) P-values are reported.  *Inflammatory biomarkers (38) highlighted in orange are associated robustly in 2011 follow-up. P-values highlighted in yellow are statistically significant. ^#^ Three models were considered with model 1 adjusting only for chronological age and sex, model 2 adjusting additionally for BMI, smoking status, socioeconomic status, alcohol use and physical activity (MET), and model 3 adjusting for all previously mentioned covariates and cell proportions. All 2018 analyses were additionally adjusted for a technical covariates “batches”, representing the two batches of DNA methylation profiling and different versions of EPIC arrays. ^##^ **Combined score:** The combined score estimating systemic inflammation is a sum of five log-transformed inflammatory biomarkers (CRP, Eotaxin, IL-18, IL2-r𝛼, HGF). | | | | | | | | | | | | | | | | | | |

# **References**

Martin J. Aryee, Andrew E. Jaffe, Hector Corrada-Bravo, Christine Ladd-Acosta, Andrew P. Feinberg, Kasper D. Hansen, Rafael A. Irizarry, Minfi: a flexible and comprehensive Bioconductor package for the analysis of Infinium DNA methylation microarrays, Bioinformatics, Volume 30, Issue 10, May 2014, Pages 1363–1369, <https://doi.org/10.1093/bioinformatics/btu049>

Timothy J. Triche, Daniel J. Weisenberger, David Van Den Berg, Peter W. Laird, Kimberly D. Siegmund, Low-level processing of Illumina Infinium DNA Methylation BeadArrays, Nucleic Acids Research, Volume 41, Issue 7, 1 April 2013, Page e90, <https://doi.org/10.1093/nar/gkt090>

Zhou W, Triche TJ Jr, Laird PW, Shen H. SeSAMe: reducing artifactual detection of DNA methylation by Infinium BeadChips in genomic deletions. Nucleic Acids Res. 2018 Nov 16;46(20):e123.
